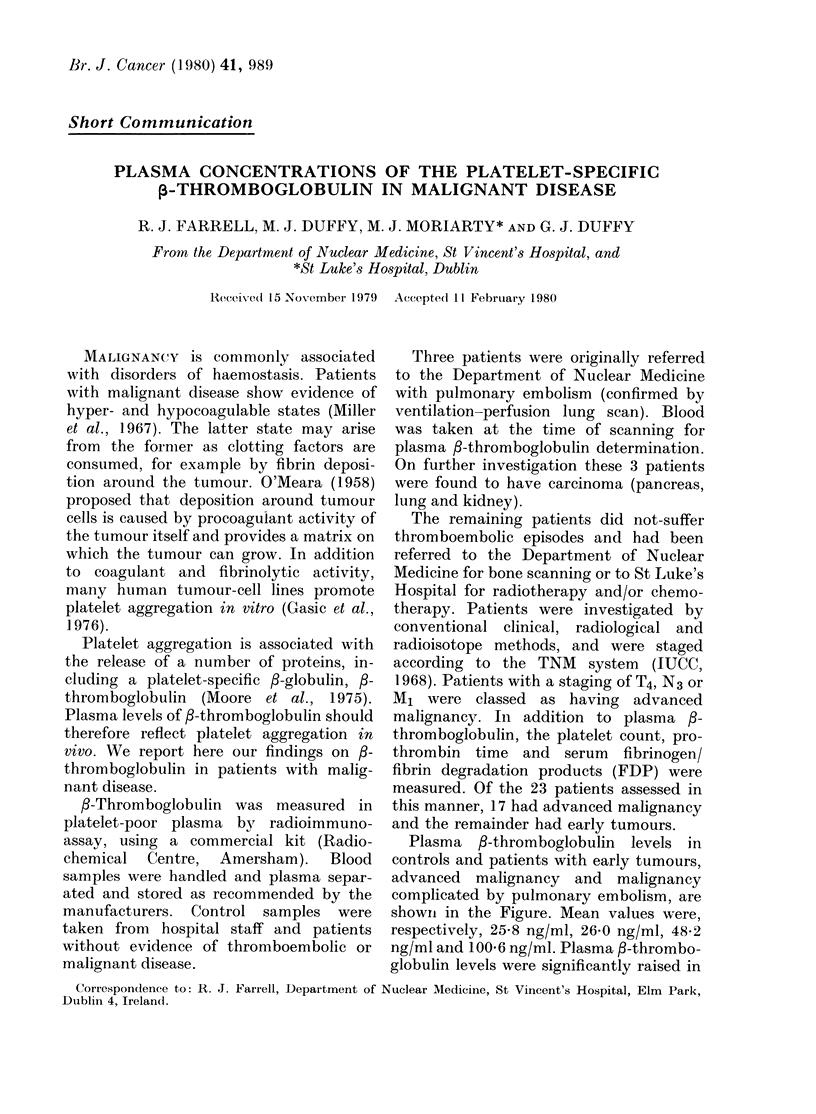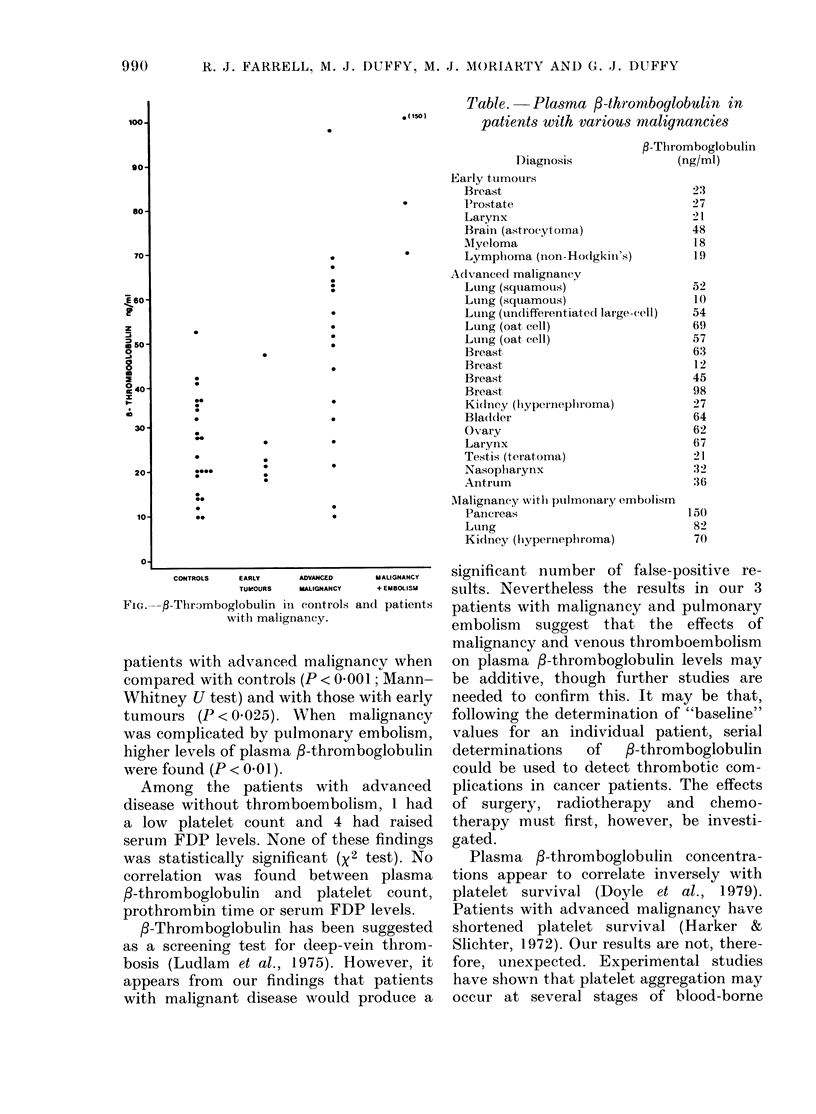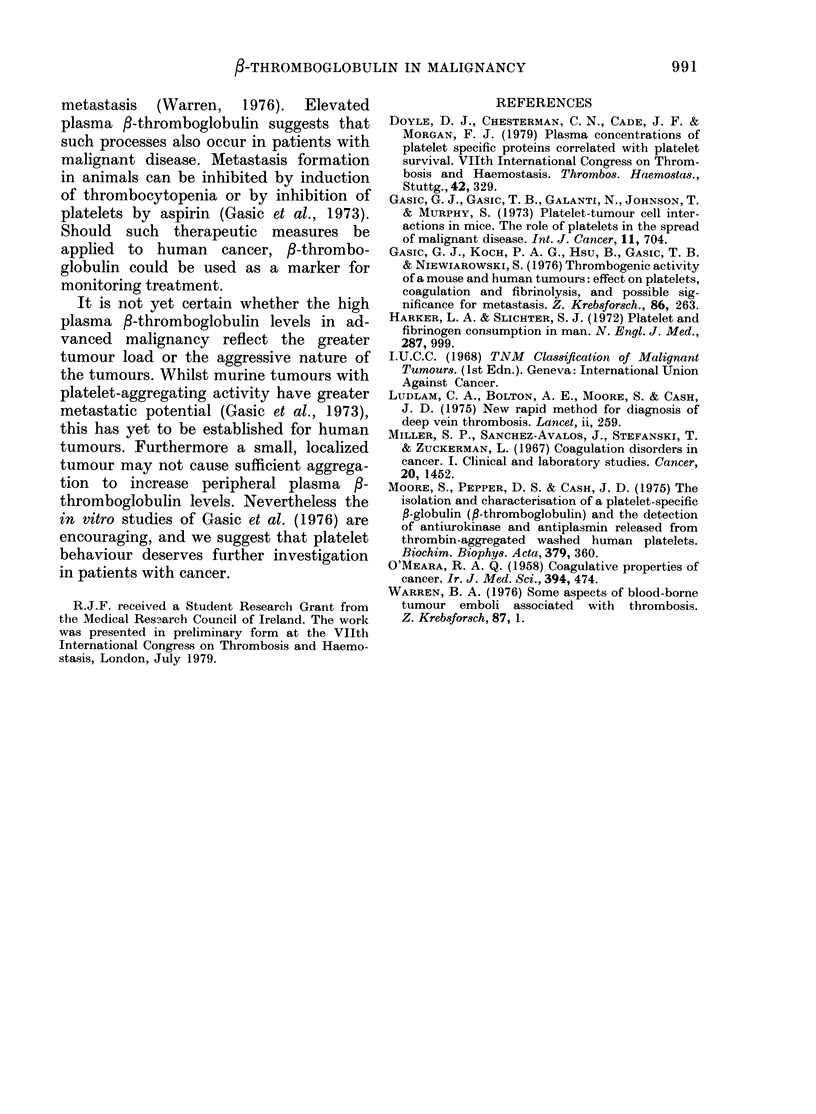# Plasma concentrations of the platelet-specific beta-thromboglobulin in malignant disease.

**DOI:** 10.1038/bjc.1980.179

**Published:** 1980-06

**Authors:** R. J. Farrell, M. J. Duffy, M. J. Moriarty, G. J. Duffy


*Br. J. Cancer* (1980) **41**, 989

***Short Communication***

### PLASMA CONCENTRATIONS OF THE PLATELET-SPECIFIC β-THROMBOGLOBULIN IN MALIGNANT DISEASE

R. J. FARRELL, M. J. DUFFY, M. J. MORIARTY* AND G. J. DUFFY

*From the Department of Nuclear Medicine, St Vincent's Hospital, and*
**St Luke's Hospital, Dublin*

Received 15 November 1979 Accepted 11 February 1980

MALIGNANCY is commonly associated with disorders of haemostasis. Patients with malignant disease show evidence of hyper- and hypocoagulable states (Miller *et al.*, 1967). The latter state may arise from the former as clotting factors are consumed, for example by fibrin deposition around the tumour. O'Meara (1958) proposed that deposition around tumour cells is caused by procoagulant activity of the tumour itself and provides a matrix on which the tumour can grow. In addition to coagulant and fibrinolytic activity, many human tumour-cell lines promote platelet aggregation *in vitro* (Gasic *et al.*, 1976).

Platelet aggregation is associated with the release of a number of proteins, including a platelet-specific β-globulin, β-thromboglobulin (Moore *et al.*, 1975). Plasma levels of β-thromboglobulin should therefore reflect platelet aggregation *in vivo*. We report here our findings on β-thromboglobulin in patients with malignant disease.

β-Thromboglobulin was measured in platelet-poor plasma by radioimmunoassay, using a commercial kit (Radiochemical Centre, Amersham). Blood samples were handled and plasma separated and stored as recommended by the manufacturers. Control samples were taken from hospital staff and patients without evidence of thromboembolic or malignant disease.

Three patients were originally referred to the Department of Nuclear Medicine with pulmonary embolism (confirmed by ventilation–perfusion lung scan). Blood was taken at the time of scanning for plasma β-thromboglobulin determination. On further investigation these 3 patients were found to have carcinoma (pancreas, lung and kidney).

The remaining patients did not-suffer thromboembolic episodes and had been referred to the Department of Nuclear Medicine for bone scanning or to St Luke's Hospital for radiotherapy and/or chemotherapy. Patients were investigated by conventional clinical, radiological and radioisotope methods, and were staged according to the TNM system (IUCC, 1968). Patients with a staging of $T_4$, $N_3$ or $M_1$ were classed as having advanced malignancy. In addition to plasma β-thromboglobulin, the platelet count, prothrombin time and serum fibrinogen/fibrin degradation products (FDP) were measured. Of the 23 patients assessed in this manner, 17 had advanced malignancy and the remainder had early tumours.

Plasma β-thromboglobulin levels in controls and patients with early tumours, advanced malignancy and malignancy complicated by pulmonary embolism, are shown in the Figure. Mean values were, respectively, 25·8 ng/ml, 26·0 ng/ml, 48·2 ng/ml and 100·6 ng/ml. Plasma β-thromboglobulin levels were significantly raised in

Correspondence to: R. J. Farrell, Department of Nuclear Medicine, St Vincent's Hospital, Elm Park, Dublin 4, Ireland.

FIG.—β-Thromboglobulin in controls and patients with malignancy.

*Table.—Plasma β-thromboglobulin in patients with various malignancies*

| Diagnosis | β-Thromboglobulin (ng/ml) |
|---|---|
| Early tumours | |
| Breast | 23 |
| Prostate | 27 |
| Larynx | 21 |
| Brain (astrocytoma) | 48 |
| Myeloma | 18 |
| Lymphoma (non-Hodgkin's) | 19 |
| Advanced malignancy | |
| Lung (squamous) | 52 |
| Lung (squamous) | 10 |
| Lung (undifferentiated large-cell) | 54 |
| Lung (oat cell) | 69 |
| Lung (oat cell) | 57 |
| Breast | 63 |
| Breast | 12 |
| Breast | 45 |
| Breast | 98 |
| Kidney (hypernephroma) | 27 |
| Bladder | 64 |
| Ovary | 62 |
| Larynx | 67 |
| Testis (teratoma) | 21 |
| Nasopharynx | 32 |
| Antrum | 36 |
| Malignancy with pulmonary embolism | |
| Pancreas | 150 |
| Lung | 82 |
| Kidney (hypernephroma) | 70 |

patients with advanced malignancy when compared with controls ($P < 0{\cdot}001$; Mann–Whitney $U$ test) and with those with early tumours ($P < 0{\cdot}025$). When malignancy was complicated by pulmonary embolism, higher levels of plasma β-thromboglobulin were found ($P < 0{\cdot}01$).

Among the patients with advanced disease without thromboembolism, 1 had a low platelet count and 4 had raised serum FDP levels. None of these findings was statistically significant ($\chi^2$ test). No correlation was found between plasma β-thromboglobulin and platelet count, prothrombin time or serum FDP levels.

β-Thromboglobulin has been suggested as a screening test for deep-vein thrombosis (Ludlam *et al.*, 1975). However, it appears from our findings that patients with malignant disease would produce a significant number of false-positive results. Nevertheless the results in our 3 patients with malignancy and pulmonary embolism suggest that the effects of malignancy and venous thromboembolism on plasma β-thromboglobulin levels may be additive, though further studies are needed to confirm this. It may be that, following the determination of "baseline" values for an individual patient, serial determinations of β-thromboglobulin could be used to detect thrombotic complications in cancer patients. The effects of surgery, radiotherapy and chemotherapy must first, however, be investigated.

Plasma β-thromboglobulin concentrations appear to correlate inversely with platelet survival (Doyle *et al.*, 1979). Patients with advanced malignancy have shortened platelet survival (Harker & Slichter, 1972). Our results are not, therefore, unexpected. Experimental studies have shown that platelet aggregation may occur at several stages of blood-borne metastasis (Warren, 1976). Elevated plasma β-thromboglobulin suggests that such processes also occur in patients with malignant disease. Metastasis formation in animals can be inhibited by induction of thrombocytopenia or by inhibition of platelets by aspirin (Gasic *et al.*, 1973). Should such therapeutic measures be applied to human cancer, β-thromboglobulin could be used as a marker for monitoring treatment.

It is not yet certain whether the high plasma β-thromboglobulin levels in advanced malignancy reflect the greater tumour load or the aggressive nature of the tumours. Whilst murine tumours with platelet-aggregating activity have greater metastatic potential (Gasic *et al.*, 1973), this has yet to be established for human tumours. Furthermore a small, localized tumour may not cause sufficient aggregation to increase peripheral plasma β-thromboglobulin levels. Nevertheless the *in vitro* studies of Gasic *et al.* (1976) are encouraging, and we suggest that platelet behaviour deserves further investigation in patients with cancer.

R.J.F. received a Student Research Grant from the Medical Research Council of Ireland. The work was presented in preliminary form at the VIIth International Congress on Thrombosis and Haemostasis, London, July 1979.

### REFERENCES

DOYLE, D. J., CHESTERMAN, C. N., CADE, J. F. & MORGAN, F. J. (1979) Plasma concentrations of platelet specific proteins correlated with platelet survival. VIIth International Congress on Thrombosis and Haemostasis. *Thrombos. Haemostas.*, Stuttg., **42**, 329.

GASIC, G. J., GASIC, T. B., GALANTI, N., JOHNSON, T. & MURPHY, S. (1973) Platelet-tumour cell interactions in mice. The role of platelets in the spread of malignant disease. *Int. J. Cancer*, **11**, 704.

GASIC, G. J., KOCH, P. A. G., HSU, B., GASIC, T. B. & NIEWIAROWSKI, S. (1976) Thrombogenic activity of a mouse and human tumours: effect on platelets, coagulation and fibrinolysis, and possible significance for metastasis. *Z. Krebsforsch.*, **86**, 263.

HARKER, L. A. & SLICHTER, S. J. (1972) Platelet and fibrinogen consumption in man. *N. Engl. J. Med.*, **287**, 999.

I.U.C.C. (1968) *TNM Classification of Malignant Tumours.* (1st Edn.). Geneva: International Union Against Cancer.

LUDLAM, C. A., BOLTON, A. E., MOORE, S. & CASH, J. D. (1975) New rapid method for diagnosis of deep vein thrombosis. *Lancet*, ii, 259.

MILLER, S. P., SANCHEZ-AVALOS, J., STEFANSKI, T. & ZUCKERMAN, L. (1967) Coagulation disorders in cancer. I. Clinical and laboratory studies. *Cancer*, **20**, 1452.

MOORE, S., PEPPER, D. S. & CASH, J. D. (1975) The isolation and characterisation of a platelet-specific β-globulin (β-thromboglobulin) and the detection of antiurokinase and antiplasmin released from thrombin-aggregated washed human platelets. *Biochim. Biophys. Acta*, **379**, 360.

O'MEARA, R. A. Q. (1958) Coagulative properties of cancer. *Ir. J. Med. Sci.*, **394**, 474.

WARREN, B. A. (1976) Some aspects of blood-borne tumour emboli associated with thrombosis. *Z. Krebsforsch*, **87**, 1.